# Residential exposure to electromagnetic fields and risk of amyotrophic lateral sclerosis: a dose–response meta-analysis

**DOI:** 10.1038/s41598-021-91349-2

**Published:** 2021-06-07

**Authors:** Tommaso Filippini, Elizabeth E. Hatch, Marco Vinceti

**Affiliations:** 1 CREAGEN-Environmental, Genetic and Nutritional Epidemiology Research Center, Department of Biomedical, Metabolic and Neural Sciences, University of Modena and Reggio Emilia, 41125 Modena, Italy; 2 Department of Epidemiology, Boston University School of Public Health, Boston, MA 02118 USA

**Keywords:** Environmental impact, Epidemiology, Amyotrophic lateral sclerosis

## Abstract

Amyotrophic lateral sclerosis (ALS) is neurodegenerative disease characterized by a fatal prognosis and still unknown etiology. Some environmental risk factors have been suggested, including exposure to magnetic fields. Studies have suggested positive associations in occupationally-exposed populations, but the link with residential exposure is still debated as is the shape of such relation. Due to recent availability of advanced biostatistical tools for dose–response meta-analysis, we carried out a systematic review in order to assess the dose–response association between ALS and residential exposure to magnetic fields. We performed an online literature searching through April 30, 2021. Studies were included if they assessed residential exposure to electromagnetic fields, based either on distance from overhead power lines or on magnetic field modelling techniques, and if they reported risk estimates for ALS. We identified six eligible studies, four using distance-based and one modelling-based exposure assessment, and one both methods. Both distance-based and particularly modelling-based exposure estimates appeared to be associated with a decreased ALS risk in the highest exposure category, although estimates were very imprecise (summary RRs 0.87, 95% CI 0.63–1.20, and 0.27, 95% CI 0.05–1.36). Dose–response meta-analysis also showed little association between distance from power lines and ALS, with no evidence of any threshold. Overall, we found scant evidence of a positive association between residential magnetic fields exposure and ALS, although the available data were too limited to conduct a dose–response analysis for the modelled magnetic field estimates or to perform stratified analyses.

## Introduction

Amyotrophic lateral sclerosis (ALS) is a rare and progressive neurodegenerative disease with still unknown etiology^1^. Across Europe, ALS prevalence has been estimated between 7 and 9/100,000 persons with an annual incidence between 1 and 3 cases/100,000^2–4^. Major advances in ALS genetics have identified more than 30 genes which confer an increased risk of the disease and likely account for 5–10% of all cases^1^. In particular, mutations in four genes (*C9orf72*, *TARDBP*, *SOD1*, and *FUS*) account for up to 70% of all familial ALS cases and 10% of sporadic form^5^. Nonetheless, some of the implicated genes are incompletely penetrant, thus genotype does not necessarily predict phenotype due to presence of oligogenic inheritance and genetic pleiotropy^1^. For these reasons, ALS is considered a complex disorder with interactions between genetic and environmental determinants^6–9^. Several occupational and environmental determinants of ALS have been proposed, encompassing biological, chemical and physical risk factors^10–16^, including exposure to magnetic fields^8,17^. In particular, previous studies have suggested a higher risk of ALS in occupationally-exposed populations^18^, but limited evidence has been provided for residential exposure to magnetic fields. In addition, individual studies have been small and have not investigated whether the association may be non-linear or have a lower threshold of effect^17^.

Given the recent availability of advanced biostatistical random-effects models for dose–response pooling of study results^19^, we carried out a systematic review in order to assess the dose–response relation between magnetic fields and ALS risks.

## Results

**Figure 1:**
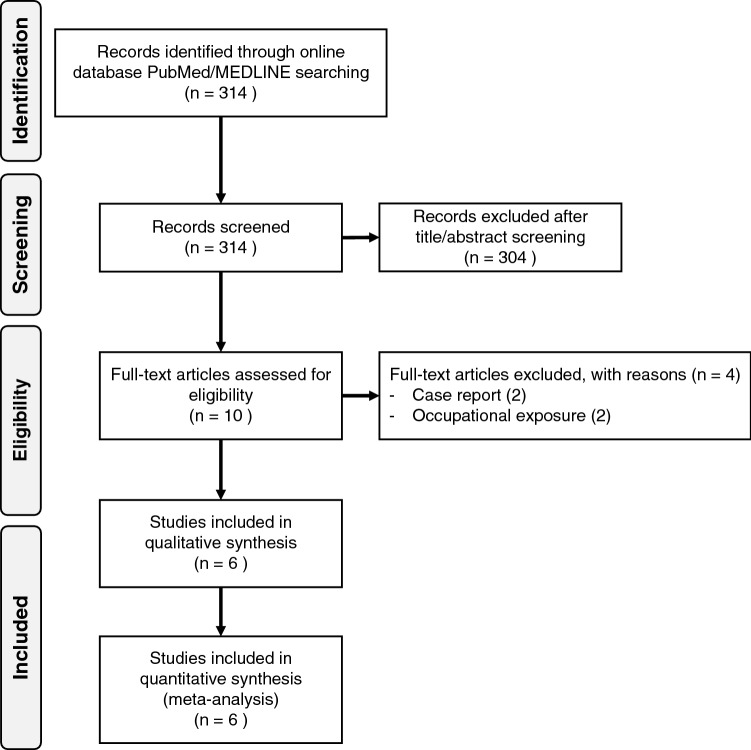
PRISMA flow-chart for study identification and selection.

Figure 1 presents PRISMA flow-chart of study identification. Out of total 314 retrieved studies, we excluded 304 studies after title and abstract screening, and further four were excluded after full text evaluation. Overall, six studies eventually fulfilled the inclusion criteria^20–25^.

Five of the included studies had a case–control design^20,21,23–25^ and one was a cohort study^22^ (Table 1). Case identification methods was based on presence of an ALS Disease Register in most of the studies^20,21,24,25^. Nonetheless, all included studies used reliable data sources to identify ALS cases based on International Disease Classification (ICD), e.g. hospital discharge records^20,21,24,25^, drug prescriptions^20,25^, or death certificate linkage^22,23^. All six studies estimated electromagnetic field exposure by calculating residential distance from power lines, and two also performed modelling-based assessment through evaluation of magnetic field intensity^23,25^. None of the included studies was judged at high risk of bias (Supplemental Table S1), though two were at moderate risk of bias due to exposure assessment which partially relied on self-report^20^, and due to lack of adjustment for confounding for some estimates^23^. In particular, although all studies implemented a multivariable model in the analysis, only two studies^21,22^ accounted for several confounding factors, while the remaining four studies had limited control for confounders. In addition, all studies had very imprecise estimates, with no clear association in either distance-based or modelling-based methods. Conversely, a strength of all included studies was the use of individual information and accurate address information for determination of the geographical coordinates and exposure assessment.

**Table 1:** Characteristics of includes studies.

Reference, year	Country, period	Design, population	Exposure assessment	Outcome assessment		Risk estimate (95% CI)	Model
Filippini 2020^20^	Italy 2008–2011 2002–2012	Case–control 95/135	Distance (m) from overhead power lines (kV not specified) using address of residence at the time of diagnosis stable for minimum 5 years	Register based (HDR, DR, drug prescription)	≥ 600 200 to < 600 50 to < 200 < 50	Referent 4.4 (0.4 − 45.9) 11.2 (1.3 − 98.4) 1.3 (0.4 − 4.6)	Adjusted by sex, age, and educational attainment
Frei 2013^21^	Denmark 1994–2010	Case–control 2990^a^/14,996	Distance (m) from power lines (132–400 kV) using cumulative duration of exposure in 5–20 years according residential history	Register-based (HDR, ICD 10 G12)	≥ 600 200 to < 600 50 to < 200 < 50	Referent 0.97 (0.81–1.16) 0.94 (0.66–1.32) 0.80 (0.34–1.89)	Matched by sex and birth date, and adjusted by disposable income, education, urbanization, no. of floors in the residential building, and marital status
Huss 2009^22^	Switzerland 2000–2005	Cohort 744/4.65 million	Distance (m) from power lines (220–380 kV) using census residential address	Fatal cases (ICD 10 G12.2)	≥ 600 200 to < 600 50 to < 200 < 50	Referent 0.72 (0.52–1.00) 0.85 (0.46–1.59) –	Age used as the underlying timescale and adjusted by sex, educational level, occupational attainment, urban–rural area, civil status, language region, no. of apartments per building, and living within 50 m of a major road
Marcilio 2011^23^	Brazil 2001–2005	Case–control 367/4706	Magnetic fields exposure (µT) from power lines (88–440 kV) using residential address from death certificate	Fatal cases (ICD 10 G12.2)	≤ 0.1 > 0.1 to ≤ 0.3 > 0.3	Referent – 0.27 (0.01–1.62) ^b^	Adjusted by race, schooling and marital status
			Distance (m) from power lines (88–440 kV) using residential address from death certificate		> 400 > 200 to ≤ 400 > 100 to ≤ 200 > 50 to ≤ 100 ≤ 50	Referent 1.24 (0.83–1.86) 1.14 (0.65–2.02) 0.49 (0.15–1.56) 0.26 (0.06–1.05) ^c^	
Seelen 2014^24^	The Netherlands 2006–2013	Case–control 1139/2864	Distance (m) from power line—high-voltage (50–150 kV) using lifetime residential history	Register-based (HDR)	≥ 600 200 to < 600 50 to < 200 < 50	Referent 1.31 (0.79–2.18) 0.73 (0.15–3.50) -	Matched by age and sex
			Distance (m) from power line—very high voltage (220–380 kV) using lifetime residential history		≥ 600 200 to < 600 50 to < 200 < 50	Referent 0.89 (0.69–1.14) 0.91 (0.60.1.37) 1.05 (0.40–2.75)	
Vinceti 2017^25^	Italy 1998–2011	Case–control 703/2737	Magnetic fields exposure (µT) from power lines (132–380 kV) using both address of residence at the time of diagnosis and 20-year stable address of residence	Register-based (HDR, DR, drug prescription)	< 0.1 0.1 to < 0.2 0.2 to < 0.4 ≥ 0.4	Referent 0.64 (0.14–2.85) 1.17 (0.32–4.26) 0.27 (0.04–2.13)	Matched by age, sex, and province of residence
			Distance (m) from power lines (132–380 kV) using both address of residence at the time of diagnosis and 20-year stable address of residence		≥ 600 200 to < 600 50 to < 200 ≤ 50	Referent 0.72 (0.56 − 0.92) 0.95 (0.67 − 1.34) 1.01 (0.53 − 1.94)	

*DR* disease registry, *HDR* hospital discharge registry, *HR* hazard ratio, *ICD* International Classification of Diseases, *OR* odds ratio.

^a^Motor neuron disease.

^b^Computed from crude data using *cci* routine in Stata 16.1 (StataCorp. College Station, TX).

^c^Risk estimate from crude model, corresponding figure from adjusted model not reported.

**Figure 2:**
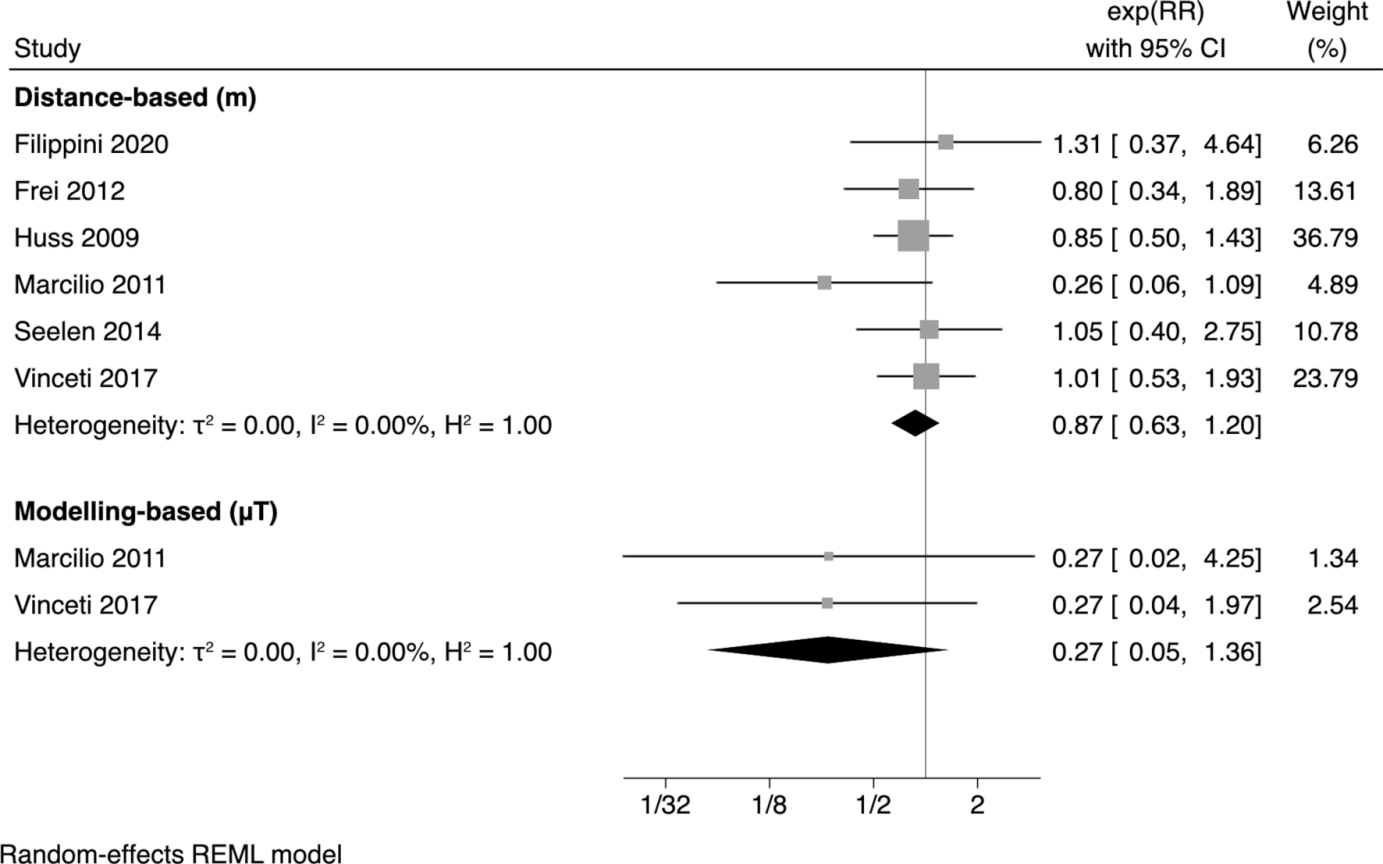
Forest plot with meta-analysis of the highest versus the lowest exposure to magnetic field, using both distance-based or modelling based-methods.

Figure 2 presents summary estimates of the meta-analysis by comparing the highest versus the lowest magnetic field exposure. Both distance-based and particularly modelling-based exposure summary estimates appear to show no excess risk for ALS, since the summary RRs comparing highest to lowest exposure categories were below unity (0.87, 95% CI 0.63–1.20, and 0.27, 95% CI 0.05–1.36, respectively) although they are highly imprecise. Stratified analysis according to method of case identification (ALS registries vs. mortality from death certificates) showed almost identical results for studies modelling-based (Supplemental Figure S1). Conversely when distance was used for exposure assessment, we found no change in ALS risk associated with magnetic field exposure in registry-based studies (summary RR 0.99, 95% CI 0.64–1.52), while risk appeared to decrease in the studies based on mortality (summary RR 0.57, 95% CI 0.19–1.71) (Supplemental Figure S2). However, the interpretation of such results is hampered by the limited number of studies in subgroup analysis and therefore the high imprecision of estimates.

The funnel-plot shows a somewhat asymmetric distribution and the result of the Egger’s test carried out on the five distance-based studies suggests unimportant to moderate small-study effects (intercept − 0.74, 95% CI − 3.16 to 1.67) (Supplemental Figure S3). Also trim-and-fill analysis shows limited evidence of small-study bias, with overall estimate of observed plus imputed data of 0.91 (95% CI 0.67–1.24).

**Figure 3:**
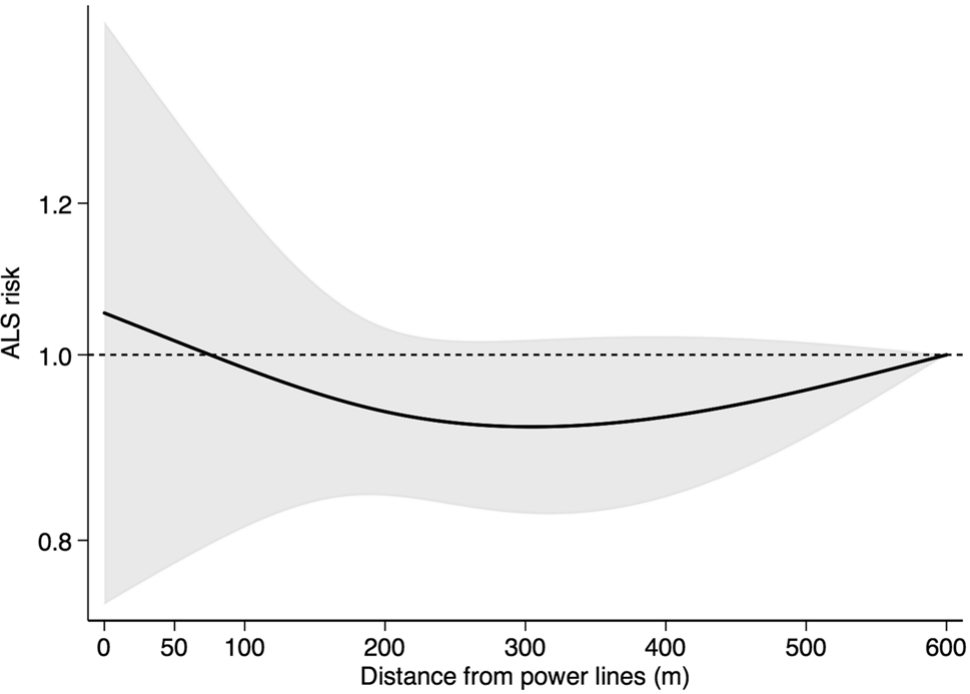
Dose–response meta-analysis of ALS risk according to decreasing residential distance from power lines.

Only two studies had estimates based on magnetic field modelling, thus it was not possible to conduct a dose-response meta-analysis for magnetic field exposure. Figure 3 presents results of dose–response meta-analysis based on distance to power lines and suggests little association with ALS. In the sensitivity analysis showing single-study effects, high variation can be noted, with some studies suggesting a slight decrease in risk among participants living closer to power lines while other studies show a small increase (Supplemental Figure S4).

## Discussion

This review reports for the first time the dose–response relation between residential exposure to magnetic fields and risk of ALS, indicating little evidence of such association. In contrast, previous studies of occupational exposure suggested a positive association with ALS^13,26–28^, especially among ‘electric workers’ such as welders, telephone or radio/television repairmen and installers, electric line installers, power-production plant operators, sewing-machine operators, and aircraft pilots, due to their exposure to low- and extremely low-frequency magnetic fields^29,30^. In addition, a recent study reported a positive association with residential exposure to ultra-high frequency magnetic fields emitted from telephone communication antennas using a model based on both their distance and power^31^.

A possible explanation of the contrasting findings between residential and occupational exposure may be due to different exposure patterns, specifically the intensity and frequency of magnetic fields experienced by workers compared to the general population. However, in most of the occupational settings considered at ‘high exposure’ the average fields measured were no more than one order of magnitude higher than those measured in residential settings^32^. Additionally, in ‘residential’ studies, spatial and temporal variability in magnetic field levels might have hampered the reliability of exposure assessment resulting in non-differential exposure misclassification and bias to the null^33^. In particular, subjects might have experienced varying magnetic fields intensity depending on the size of their house, presence of any shielding material in the building, or amount of time spent at home compared to other places of living or working. Most studies assessed residential history^21,22,24,25^, but only two studies took into account residential mobility in the analysis^21,22^, and two studies measured magnetic fields near the residence at the time of death as opposed to before diagnosis^20,23^. Three studies investigated the association in subjects with a stable residence^21,22,25^. In a study in Denmark, after assessment of cumulative duration of residency within a distance of 50 m, magnetic fields did not increase risk of motor neuron disease in subjects considered most exposed^21^. In a Swiss study, when the analysis was restricted to individuals living > 15 years at the same residence before diagnosis, the results showed little change in ALS risk, compared with results in all subjects^22^. In our previous study, we found an increased ALS risk in the intermediate category only (0.2 to  < 0.4 µT) among subjects who were residentially stable, although characterized by high statistical imprecision (OR 2.02, 95% CI 0.18–22.53)^25^. In the sensitivity analysis showing single-study effects, we noted a high variation possibly linked to different susceptibility to magnetic fields among study populations, thus our analysis does not enable us to rule out entirely positive associations in selected subgroups and at very high exposure. Finally, we also cannot rule out the occurrence of residual confounding, since only two studies, showing little association with ALS, reported risk estimates for magnetic fields adjusted for several other potential environmental risk factors^21,22^, such as air pollution using urbanization levels or distance to major roads, in the models.

Interestingly, it has been suggested that the increased risk of ALS in some occupations, especially machinery operators and drivers, might be linked to diesel exhaust rather than magnetic field exposure^34^. Unfortunately, only a few studies investigated environmental exposure to outdoor air pollutants and ALS. In particular, the long-term exposure to PM_2.5_, NO_x_ and NO_2_ air pollutants showed a positive association with ALS risk in highly exposed subjects in both the Netherlands and Spain^14,35^. Similarly, high levels of residential exposure to traffic-derived aromatic solvents has been associated with increased risk of ALS in a U.S. study^36^. However, in a recent case–control we carried out in Italy, we did not find a positive association between PM_10_ exposure and ALS, except for a very imprecise increase in risk between 10 and 20 µg/m^3^ of annual maximum PM_10_ levels^11^. In addition, an interaction between magnetic fields and air pollutants has been proposed due to formation of charged corona ions produced in the vicinity of power lines^37,38^. In particular, corona ions may interact with aerosol particles by modification of the electric charge state of air pollutants^38^. It has been supposed that charged air pollutants may have an increased probability of deposition on the skin and in the respiratory system, thus leading to potential increased risk for human health, including disturbances in circadian rhythm and also cancer^39,40^. The transportation of charged airborne particles at long distances from the power lines by the wind^37^ might also explain the lack of a dose–response association with increasing exposure to magnetic fields, as well as the inconsistent positive association for subjects in the intermediate category but not for those living closest to power lines as shown in several studies^20,23,25^. Finally, we cannot rule out confounding by occupational exposure to magnetic fields. Although some studies combined residential and occupational magnetic field exposure to reduce misclassification, a direct relation between the two measurements was not assessed^41–43^.

Laboratory studies provide some biological plausibility of the positive association between magnetic fields exposure and ALS. Low-frequency magnetic exposure may act as a risk factor for the occurrence of oxidative stress-based nervous system pathologies associated with ageing in an animal model^44^. In particular, an enhancement in SOD-2 dismutase activity has been reported in young animals, while aged animals underwent a major weakening of antioxidant defense systems. Similarly, another animal study using extremely low-frequency magnetic fields suggested harmful neurological effects due to development of lipid peroxidation, especially to the basal forebrain and frontal cortex^45^. An in vitro ALS model reported that magnetic field exposure caused impairment of iron homeostasis in SOD-1 mutant cells through deregulation of expression of iron-related genes, recently suggested as molecular determinant in the pathogenesis of ALS^46^. However, in mouse models expressing mutant Cu/Zn-superoxide dismutase, low-frequency magnetic field exposure did not alter disease onset and survival^47^. Another report implementing a SOD-1 transgenic mouse model did not reveal any effect on survival between exposed and unexposed groups. However, slightly worse motor function occurred in the experimental groups during magnetic fields exposure period, although the differences were very imprecise^48^. Despite these null findings, it should be noted that the mouse SOD-1 models would correspond to familial rather than sporadic ALS. This may explain the contrasting results from animal and in vitro studies, and also possibly indicate differential effects on the two ALS forms.

Some limitations of our study should be noted. Despite re-analysis of previous studies in order to include more data, a small sample size limited the interpretation of our findings. In addition, the low number of studies did not allow dose–response analysis for modelling-based studies. We also cannot rule out the occurrence of residual confounding since only two included studies took into account a large number of potential confounders in the multivariable models^21,22^, while some studies took into account some established or putative risk factors such as socio-economic status and educational attainment^49,50^, smoking^51^, residential exposure to pesticides^52,53^, or raw water^10,54^. Finally, although results of Egger’s test and trim-and-fill analysis suggest limited evidence of small-study bias, the slight asymmetric distribution of funnel-plots may indicate some publication bias.

## Conclusions

Overall, we found little association between exposure to magnetic fields and risk of ALS, using either distance from high-voltage overhead power lines or magnetic field modelling, although the available data were too limited to conduct a dose–response analysis for the modelled exposure studies or to perform further stratified analyses. Therefore, possible associations between magnetic fields exposure and ALS risk in selected subgroups and at very high exposure cannot be entirely ruled out.

## Methods

### Literature search

We performed a systematic according to the Preferred Reporting Items for Systematic Reviews and Meta-Analyses (PRISMA) guidelines^55^. We carried out literature search in Pubmed/MEDLINE online database since its inception until April 30, 2021, without language restrictions for the studies. The research question was configured according to PECOS statement (Population, Exposure, Comparator(s), Outcomes, and Study design): “Is residential exposure to electromagnetic fields, as assessed through overhead power lines, positively associated with risk of amyotrophic lateral sclerosis in nonexperimental studies, also taking into account the different levels of exposure?”^56^. Accordingly, we used search terms related to “amyotrophic lateral sclerosis” and “electromagnetic fields” or “overhead power lines”. Detailed search terms are reported in Supplemental Table S2. We further used citation chasing techniques (e.g. reference list scanning of included studies and of previous reviews, backward/forward citations) to identify further relevant papers^57^. Inclusion criteria were: assessment of residential exposure to electromagnetic fields, based either on distance from high-voltage overhead power lines or on magnetic field modelling techniques; reporting of risk estimates for ALS, along with their 95% confidence intervals, or availability of enough data to calculate them. Two authors reviewed all titles and abstracts independently, and conflicts were solved after discussion and when needed with the help of third person.

### Data extraction

The following data were extracted from each eligible study: (1) first author name; (2) publication year; (3) location; (4) study design; (5) recruitment period; (6) number of cases and of total study population; (7) exposure assessment method of magnetic field; (8) outcome assessment method; (9) risk estimates with their 95% CIs from the most adjusted model at each level of electromagnetic field exposure; (10) adjustment variables in multivariable analysis.

### Risk of bias assessment

We assessed risk of bias of included studies using the Risk of Bias in Non-randomized Studies of Exposures (ROBINS-E) tool^58^. Two authors independently assessed seven domains: (1) bias due to confounding; (2) bias in selecting participants in the study; (3) bias in exposure classification; (4) bias due to departures from intended exposures; (5) bias due to missing data; (6) bias in outcome measurement; (7) bias in the selection of reported results. Supplemental Table S3 reports summary criteria for risk of bias evaluation. Studies were considered of overall low risk of bias if they were judged at low risk in all domains. Conversely, they were considered at overall moderate or high risk of bias, if they were judged at high risk in one or ≥ 2 domains, respectively.

### Data analysis—meta-analysis and dose–response meta-analysis

We performed a meta-analysis based on categorical exposure to magnetic field, i.e. we used the risk estimates which compared the highest versus the lowest exposure category from each study and we combined them using a restricted maximum likelihood random effects model. Analyses were stratified according to type of exposure assessment, i.e. distance to power lines and modelled magnetic field intensity. We then performed a dose–response meta-analysis using the one-stage approach to assess the shape of the relation between decreasing distance from power lines and ALS risk as already implemented in other fields^59,60^. To do that, we considered as exposure dose the midpoint of each exposure strata for the intermediate categories, while for the highest and lowest exposure categories we used a value that was 20% higher or lower than the closest boundary^61^. We used a restricted cubic spline model with 3 knots at fixed categories (50, 200, and 600 m) as they were used in almost all included studies. We used a generalized least-squares regression taking into account the correlation within each set of published effect estimates using a multivariate random-effect meta-analysis through the restricted maximum likelihood method^62,63^.

We checked for the possible presence of small-study bias using funnel plots for studies reporting highest versus lowest exposure, and performing Egger’s test^64^ and trim-and-fill analysis when at least five studies are available. We also evaluated the influence of variation across studies through the graphical overlay of study-specific predicted curves including fixed and random effects^62^. We used Stata software (v 16.1, 2021-Stata Corp., College Station, TX) for all data analyses, namely ‘*meta*’ and ‘*drmeta*’ routines.

### Data analysis—re-analysis of previous studies

For the purpose of this review, we re-analyzed two previous studies of the association between distance from overhead power-lines and ALS risk. In the first study, we used subjects from a population-based case–control study^25^ including 703 newly-diagnosed ALS cases and 2737 matched controls randomly selected from residents in four Italian provinces (Catania, Modena, Reggio Emilia, and Parma) where only modelling-based exposure to magnetic fields was performed. Using a geographical information system, we geocoded subjects’ residence at the time of case diagnosis and we measured distance from the closest high-voltage power lines (≥ 132 kV) using a methodology already presented^65^. Using a conditional logistic regression model matched by age, sex, and province of residence, we estimated ALS risk according to distance from overhead power-lines at < 50 m, between 50 and  < 200 m, between 200 and  < 600 m, using ≥ 600 m as referent. These cutpoints were selected for comparison with most of previous studies^21,22,24^. In the second re-analysis, we used data of a population-based case–control study^20^ including 95 cases and 135 randomly selected population controls carried out in four Italian provinces (Catania, Modena, Reggio Emilia, and Novara). In addition to the previous analysis assessing proximity to magnetic fields through a questionnaire by asking at which residential address subjects might have been exposed, we assessed the distance of the closest overhead power line from their home through a geographical information system and by using Google Earth Pro 7.3 software, Google LLC. We then computed ALS risk according to residential distance from power lines using the same cutpoints at 50, 200 and 600 m using an unconditional logistic model adjusted by age, sex, and educational attainment.

## Supplementary Information

Supplementary Information 1.

## Data Availability

All data generated or analyzed during this study are included in this published article and its supplementary information file.
