# Supplementary material for: Residential exposure to electromagnetic fields and risk of amyotrophic lateral sclerosis: a dose–response meta-analysis
Source: Sci Rep. 2021 Jun 7;11:11939. doi: 10.1038/s41598-021-91349-2 (PMC8185090; doi:10.1038/s41598-021-91349-2)

**SUPPLEMENTAL MATERIAL**

Title: Residential exposure to electromagnetic fields and risk of amyotrophic lateral sclerosis: A dose-response meta-analysis

Authors: Tommaso Filippini^1^, Elizabeth E. Hatch^2^, Marco Vinceti^1,2^

**Affiliations:**

^1^CREAGEN - Environmental, Genetic and Nutritional Epidemiology Research Center, Department of Biomedical, Metabolic and Neural Sciences, University of Modena and Reggio Emilia, Modena 41125, Italy;

^2^Department of Epidemiology, Boston University School of Public Health, Boston, MA 02118, USA.

**Supplemental Table S1**. Summary Risk of Bias assessment with overall study-level risk of bias.

| **Studies** | Domain (1) | Domain (2) | Domain (3) | Domain (4) | Domain (5) | Domain (6) | Domain (7) | **Overall** |
| --- | --- | --- | --- | --- | --- | --- | --- | --- |
| Filippini 2020 ^18^ | Low | Low | High | Low | Low | Low | Low | **Moderate** |
| Frei 2013 ^19^ | Low | Low | Low | Low | Low | Low | Low | **Low** |
| Huss 2009 ^20^ | Low | Low | Low | Low | Low | Low | Low | **Low** |
| Marcilio 2011 ^21^ | High | Low | Low | Low | Low | Low | Low | **Moderate** |
| Seelen 2014 ^22^ | Low | Low | Low | Low | Low | Low | Low | Low |
| Vinceti 2017 ^23^ | Low | Low | Low | Low | Low | Low | Low | Low |

**Supplemental Table S2.** Search strategy for PubMed/MEDLINE database.

| Search item | Search term |
| --- | --- |
| #1 | amyotrophic lateral sclerosis[MH] |
| #2 | amyotrophic lateral sclerosis[TIAB] |
| #3 | motor neuron diseases[MH] |
| #4 | motor neuron disease[TIAB] |
| #5 | neurodegenerative diseases[MH] |
| #6 | neurodegenerative diseases[TIAB] |
| #7 | #1 OR #2 OR #3 OR #4 OR #5 OR #6 |
| #8 | magnetic fields[MH] |
| #9 | magnetic fields[TIAN] |
| #10 | overhead power lines[TIAB] |
| #11 | power lines[TIAB] |
| #12 | high-voltage power lines[TIAB] |
| #13 | #8 OR #9 OR #10 OR #11 OR #12 |
| #14 | #7 AND #13 |
| #15 | humans[MH] |
| #16 | #14 AND #15 |

**Supplemental Table S3**. Criteria adopted for Risk of Bias assessment.

| **Domains** | **Criteria** |
| --- | --- |
| (1) Bias due to confounding | Factors considered mandatory in order to judge a study at low risk of bias are age and sex. |
| (2) Bias in selecting participants in the study | Selection of eligible participants must not be related to magnetic field exposure to consider a study at low risk. |
| (3) Bias in exposure classification | Possible misclassification for studies based on self-report for exposure assessment. |
| (4) Bias in departure from intended exposure | We considered a study at low risk of bias if exposure assignment if exposure assessment was the same for all participants and did not change over the study period |
| (5) Bias due to missing data | Studies with less than 10% are considered at low risk. |
| (6) Bias in outcome measurement | Possible bias based on the modality of outcome assessment. High risk in case of assessment based on self-report only without external validation. |
| (7) Bias in selection of reported results | Evidence that results have not been selected. Clear reporting of statistical methods. |

**Supplemental Figure S1**. Forest plot with meta-analysis of the highest versus the lowest exposure to magnetic field using modelling-based method stratified by outcome (incidence vs. mortality).

**Supplemental Figure S2**. Forest plot with meta-analysis of the highest versus the lowest exposure to magnetic field using both distance-based method stratified by outcome (incidence vs. mortality).

**Supplemental Figure S3**. Funnel-plot for small-study bias assessment according exposure assessment method. Black diamonds represent studies included in each analysis, the x-axis indicates the study effect/results through its risk ratio (RR), and the y-axis indicates study precision through its standard error. The outer dashed lines indicate the triangular region within which 95% of studies are expected to lie in the absence of both biases and heterogeneity. The solid vertical line corresponds to overall summary RR from meta-analysis of included studies

**Supplemental Figure S4.** Dose-response meta-analysis ALS risk according to decreasing residential distance from power lines. Sensitivity analysis showing single-study effects.


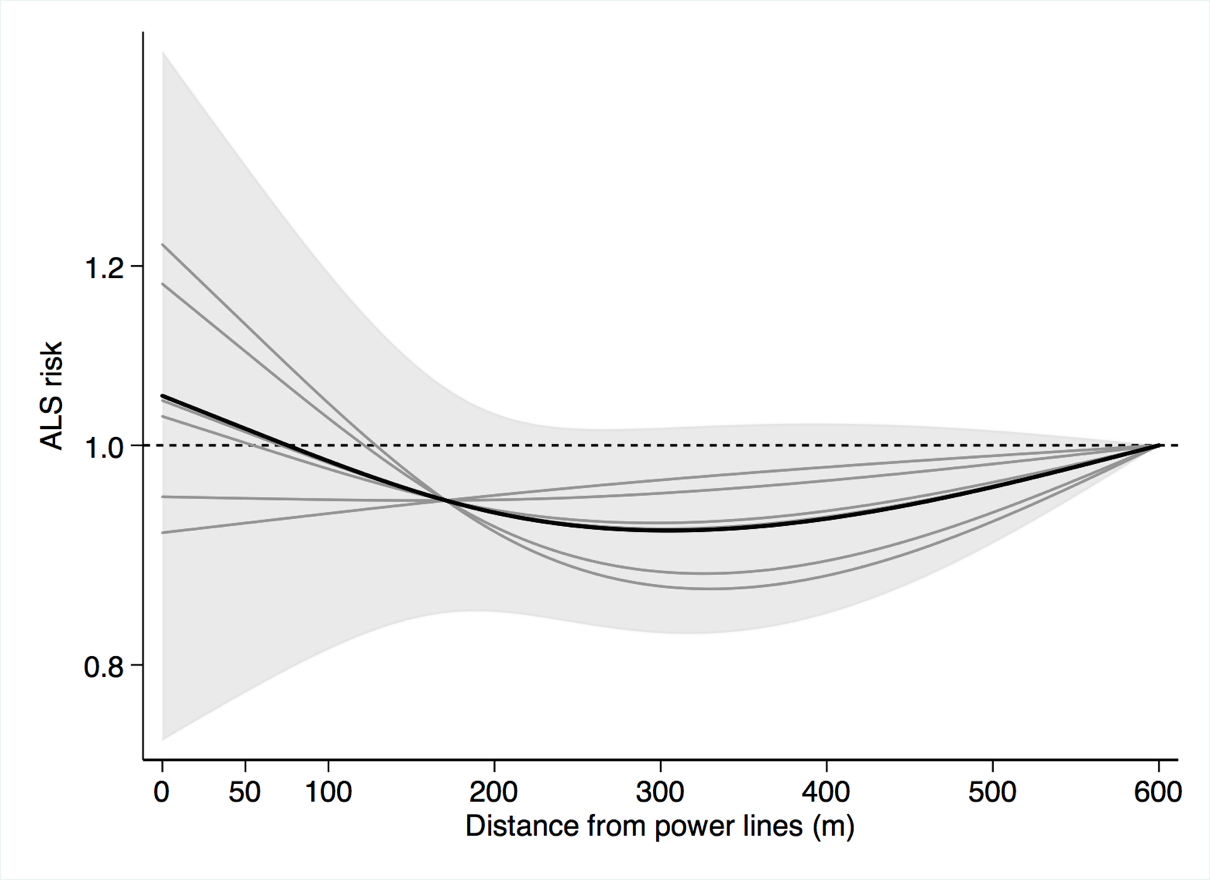

Supplement: Supplementary file 1 — Supplementary Information 1. [file 41598_2021_91349_MOESM1_ESM.docx]
